# Spatiotemporal weather forecasting via multi-scale graph neural networks and latent diffusion models

**DOI:** 10.1371/journal.pone.0348354

**Published:** 2026-06-04

**Authors:** ZhiPeng Wu

**Affiliations:** School of Finance, Hefei University of Economics, Hefei, Anhui, China; Khalifa University, UNITED ARAB EMIRATES

## Abstract

Accurate weather prediction is crucial in agriculture, disaster prevention, and public safety. Challenge: Traditional numerical models have high computational costs and struggle with atmospheric nonlinearity and chaos, while existing deep learning methods face limitations in handling spatial heterogeneity and non-Euclidean data. Solution: This paper introduces the STGLDWeather method. It combines multi-scale spatiotemporal graph neural networks (MS-ST-GNN) and latent diffusion models (LDM) to capture multi-scale spatiotemporal dependencies in weather data and model the temporal evolution of weather conditions in latent space. Conclusion: Experiments on real weather datasets show that STGLDWeather significantly outperforms existing state-of-the-art baselines in prediction accuracy and computational efficiency, particularly excelling in temperature, geopotential height, and wind speed forecasts.

## 1 Introduction

Accurate weather forecasting is of critical importance in guiding daily activities and industrial operations. The ability to predict weather elements, such as temperature, humidity, and wind speed, and long-term climate phenomena like the El Niño and tropical atmospheric oscillations [1,2], plays a key role in agriculture, disaster prevention, and public safety. Weather forecasting is typically divided into two main categories: weather element prediction, which focuses on forecasting atmospheric physical indicators in a specific region [3] at a given time, and climate phenomenon prediction, which aims to identify statistical regularities and periodic variations over longer time scales [4], ranging from several years to decades.

Traditional weather forecasting methods have relied on numerical models that solve complex physical equations. While effective, these models suffer from high computational costs and limitations in modeling the non-linear and chaotic nature of the atmosphere [5,6]. In contrast, deep learning methods have emerged as powerful tools for learning intricate patterns [7] in large-scale meteorological datasets without explicitly solving differential equations. This advantage significantly reduces computational complexity while maintaining competitive accuracy compared to traditional physics-based models [7,8].

However, current deep learning approaches face challenges in handling the spatial heterogeneity and non-Euclidean nature of weather station data. Weather data is inherently spatiotemporal, collected from sensor networks distributed across diverse geographical locations. This spatial structure is often irregular and is best represented as a graph rather than a Euclidean grid [9]. As a result, graph neural networks (GNNs) [10], which excel at modeling non-Euclidean data and capturing the complex relationships between spatial nodes, have gained increasing popularity in the domain of weather forecasting.

In this paper, we propose a method called STGLDWeather to address the challenge of improving weather forecasting accuracy by leveraging a **Multi-Scale Spatio-Temporal Graph Neural Network (MS-ST-GNN)** combined with a **Latent Diffusion Model (LDM)**. This approach is motivated by the need to handle the multi-scale nature of weather data, where both local and global spatial relationships, as well as short-term and long-term temporal dynamics, are crucial for accurate prediction. The proposed MS-ST-GNN encoder is specifically designed to extract features across various spatial and temporal scales, ensuring the effective capture of both local and global patterns. Additionally, by integrating the LDM, we introduce a novel mechanism to model the temporal evolution of weather conditions more effectively. Diffusion models, commonly used for generating data, excel at capturing complex dynamics by simulating stochastic processes. In our work, we adapt the diffusion process for spatiotemporal forecasting, allowing the LDM to model the evolution of weather conditions in a latent space while preserving rich spatiotemporal dependencies. The key contributions of our work are as follows:

- Multi-Scale Spatio-Temporal Feature Extraction: To comprehensively capture the multi-scale spatiotemporal dependencies in weather data, we introduce a novel MS-ST-GNN encoder. This encoder extracts features across multiple temporal windows and spatial scales, enabling the model to represent both fine-grained and long-term dependencies within the data.
- Latent Diffusion Process for Temporal Dynamics: We integrate a Latent Diffusion Model (LDM) to model the temporal evolution of weather conditions in a latent space. Diffusion models, widely used for their ability to capture complex stochastic processes, are applied here to spatiotemporal forecasting tasks, where they effectively model the progression of weather conditions over time.
- Comprehensive Evaluation on Real-World Data: We conduct extensive experiments on real-world meteorological datasets, demonstrating that our approach significantly outperforms state-of-the-art baselines in terms of both accuracy and computational efficiency. Our model achieves robust results even under challenging forecasting scenarios involving irregular spatial data and varying temporal scales.

## 2 Related work

### 2.1 Deep learning for weather forecasting

Deep learning has revolutionized the field of weather forecasting by leveraging large-scale data to model complex atmospheric dynamics without the need to explicitly solve differential equations. Traditional numerical weather prediction (NWP) models [11], while accurate in many respects, often suffer from high computational complexity and limitations in capturing non-linear and chaotic weather phenomena. In contrast, deep learning methods, such as convolutional neural networks (CNNs) [12,13] and recurrent neural networks (RNNs) [14], have demonstrated the ability to efficiently learn patterns from vast amounts of meteorological data.

Recent advances have seen the application of models like Long Short-Term Memory (LSTM) [15] networks and attention mechanisms, which are specifically designed to capture temporal dependencies in weather data. For instance, models such as DeepMind’s weather nowcasting system [16] have achieved remarkable success in short-term precipitation forecasting by applying deep learning techniques to radar data. However, these models primarily operate on grid-based Euclidean data, which limits their ability to represent the irregular and non-Euclidean nature of weather station networks [17]. To address this, there is a growing interest in graph-based models that can handle such complexities more naturally.

### 2.2 Spatio-temporal graph neural network

Graph neural networks (GNNs) have emerged as a powerful framework for modeling data with complex spatial dependencies, making them particularly well-suited for weather forecasting tasks. In a typical weather forecasting setup, data is collected from a network of weather stations, which can be naturally represented as nodes in a graph. The relationships between stations, influenced by geographical proximity [18] or shared weather patterns [19], form the edges. GNNs enable the modeling of these interactions, providing a flexible and effective tool for capturing spatial dependencies that are difficult to model with traditional deep learning architectures.

To capture the temporal dimension, spatio-temporal GNNs (ST-GNNs) [20,21] have been proposed, which combine graph convolutions with temporal sequence modeling techniques. For example, recent models like Spatio-Temporal Graph Convolutional Networks (ST-GCNs) and Graph Attention Networks (GATs) [22] extend GNNs to account for the evolution of weather conditions over time. These methods have shown strong performance in applications such as traffic prediction [23,24] and environmental modeling [25,26], but their adoption in weather forecasting is still in its early stages. One of the key challenges that remains is how to effectively capture dependencies across multiple temporal and spatial scales [27], which is crucial for accurate weather prediction.

### 2.3 Diffusion model

Diffusion models, traditionally used in physics and biology to simulate stochastic processes, have recently gained attention in machine learning for their ability to model complex data distributions. In particular, these models have been successfully applied to generative tasks, where they simulate the diffusion of information through a system over time. The essence of a diffusion model [28,29] is to introduce noise into a data distribution and learn how to reverse this process, gradually removing the noise to reconstruct the original data.

In the context of weather forecasting, diffusion models can be adapted to model the temporal evolution of atmospheric conditions. By mapping weather data to a latent space, the diffusion process can simulate the gradual changes in weather patterns, capturing complex temporal dependencies that may be missed by simpler models [30]. Recent advancements have explored combining diffusion models with deep learning techniques, where the diffusion process is applied in a learned latent space to enhance prediction capabilities [31]. The application of diffusion models in spatiotemporal forecasting remains a promising area of research, offering a new way to handle the inherent uncertainty and variability of weather data [4,10].

### 2.4 Neural operator learning

Beyond traditional deep learning architectures, neural operator learning has recently emerged as a powerful paradigm for solving partial differential equations (PDEs) and modeling continuous physical systems. For instance, [32] proposed non-linear operator approximations for initial value problems, capturing system dynamics by learning mappings between infinite-dimensional function spaces. Furthermore, addressing coupled physical processes, [33] introduced coupled multiwavelet operator learning for coupled differential equations, demonstrating efficacy in multi-physics modeling. While neural operators excel at approximating continuous solution operators and achieving resolution invariance, they face limitations when applied to real-world weather station observation data. First, neural operators typically assume data resides on continuous domains or regular grids, whereas weather station data is inherently discrete and irregularly distributed in space (non-Euclidean), making Graph Neural Networks (GNNs) a more natural choice for representing topological relationships. Second, most neural operators focus on deterministic mappings, yet the atmospheric system is inherently chaotic. In contrast, our proposed STGLDWeather integrates Latent Diffusion Models (LDMs) to simulate weather evolution via stochastic processes in a latent space. This generative approach not only captures multi-scale spatiotemporal dependencies but is also better suited than deterministic operators for characterizing the stochasticity and complex distributional shifts inherent in weather forecasting.

## 3 Method

### 3.1 Problem definition

**Fig 1:**
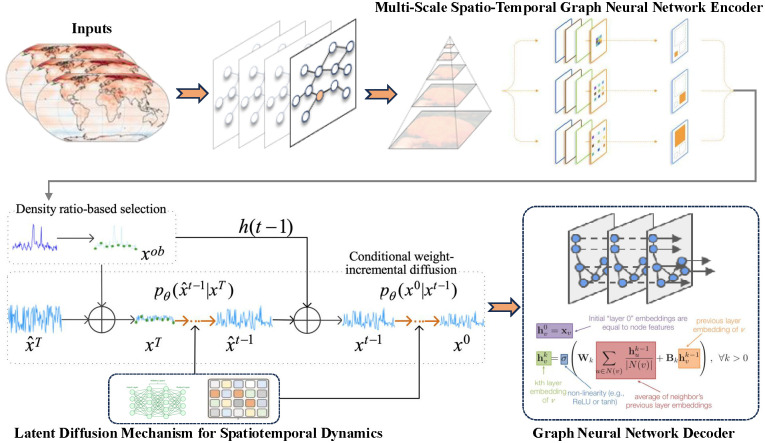
Overview of our model STGLDWeather.

In weather forecasting tasks, we typically handle data from multiple sensors located in different geographical positions. Each sensor provides various types of weather information, such as temperature, humidity, and wind speed. Suppose we have *N* sensors, with each sensor’s data represented as a *C*-dimensional feature vector. Our goal is to make accurate weather predictions based on this spatiotemporal data. Formally, we define the sensor network as a graph *G* = (*V*, *E*), where *V* is the set of nodes representing the *N* sensors, and *E* is the set of edges representing the connections between sensors. Each node $v_{i}\in V$ has a *C*-dimensional feature vector ${𝐱}_{i}\in {\mathbb{R}}^{C}$. Our problem is to predict future weather conditions given a sensor network and its historical data. To address this problem, we propose a framework that combines a Multi-Scale Spatio-Temporal Graph Neural Network (ST-GNN) with a Latent Diffusion Model (LDM). Specifically, we use the ST-GNN to extract spatiotemporal features, perform a diffusion process in the latent space through the LDM, and finally predict the weather conditions using a GNN decoder, the main figure is as shown in Fig 1.

### 3.2 Multi-scale spatio-temporal graph neural network encoder

To effectively model the multi-scale dependencies inherent in spatiotemporal data, we introduce the Multi-Scale Spatio-Temporal Graph Neural Network Encoder (MS-ST-GNN). This encoder is designed to capture features across various temporal and spatial scales [34], enhancing the model’s ability to learn complex patterns and improve prediction performance.

#### 3.2.1 Multi-scale spatio-temporal feature extraction.

Given a sensor network *G* = (*V*, *E*) with *N* nodes, where each node has a feature vector of dimension *C*, we represent the spatiotemporal data of the sensor nodes as a feature matrix $𝐗\in {\mathbb{R}}^{N\times T\times C}$, where *T* denotes the number of time steps.

We define multiple temporal scales $t_{k}\in \{t_{1},t_{2},\ldots ,t_{K}\}$, each corresponding to a distinct time window. For each temporal scale *t*_*k*_, the corresponding feature matrix ${𝐗}_{t_{k}}\in {\mathbb{R}}^{N\times C}$ is computed as:


$$\begin{array}{c} {𝐗}_{t_{k}}=\frac{1}{t_{k}}\sum_{i=1}^{t_{k}}𝐗[:,-i,:] \end{array}$$ (1)

Here, 𝐗[:,−i,:] denotes the feature matrix at the *i*-th time step, aggregated over the last *t*_*k*_ time steps to form the multi-scale temporal features.

#### 3.2.2 Multi-layer graph convolutional network.

To extract spatial features at each temporal scale *t*_*k*_, we employ a multi-layer Graph Convolutional Network. Given the adjacency matrix $𝐀\in {\mathbb{R}}^{N\times N}$ and the feature matrix ${𝐗}_{t_{k}}$, the graph convolution operation at layer *l* is defined as:


$$\begin{array}{c} {𝐇}_{t_{k}}^{\left(l+1\right)}=\sigma \left(𝐀{𝐇}_{t_{k}}^{\left(l\right)}{𝐖}_{t_{k}}^{\left(l\right)}\right) \end{array}$$ (2)

where ${𝐇}_{t_{k}}^{\left(l\right)}$ represents the node features at layer *l*, ${𝐖}_{t_{k}}^{\left(l\right)}$ is the learnab*l*e weight matrix, and σ(·) denotes the activation function. The initial input for the GCN is ${𝐇}_{t_{k}}^{\left(0\right)}={𝐗}_{t_{k}}$. After *L* layers of graph convolution, the spatial features for each temporal scale *t*_*k*_ are captured in the node feature representation ${𝐇}_{t_{k}}^{\left(L\right)}\in {\mathbb{R}}^{N\times D_{k}}$, where *D*_*k*_ is the feature dimension at scale *t*_*k*_.

#### 3.2.3 Multi-scale fusion.

To combine information from all temporal scales, we concatenate the features extracted at each scale:


$$\begin{array}{c} {𝐇}_{\text{multi}}=\text{Concat}({𝐇}_{t_{1}}^{\left(L\right)},{𝐇}_{t_{2}}^{\left(L\right)},\ldots ,{𝐇}_{t_{K}}^{\left(L\right)}) \end{array}$$ (3)

Here, ${𝐇}_{\text{multi}}\in {\mathbb{R}}^{N\times (D_{1}+D_{2}+\cdots +D_{K})}$ is the multi-scale feature representation, capturing information from all temporal scales.

To map these multi-scale features into a unified latent space, we apply a fully connected layer:


$$\begin{array}{c} 𝐇={𝐇}_{\text{multi}}{𝐖}_{\text{multi}}+{𝐛}_{\text{multi}} \end{array}$$ (4)

where ${𝐖}_{\text{multi}}\in {\mathbb{R}}^{(D_{1}+D_{2}+\cdots +D_{K})\times D}$ is the learnable weight matrix, ${𝐛}_{\text{multi}}\in {\mathbb{R}}^{D}$ is the bias vector, and $𝐇\in {\mathbb{R}}^{N\times D}$ is the final multi-scale spatiotemporal feature representation. This multi-scale feature extraction mechanism allows us to comprehensively capture the complex spatiotemporal dependencies in the data, thereby enhancing the expressiveness and robustness of the model.

### 3.3 Latent diffusion mechanism for spatiotemporal dynamics

Our latent diffusion model (LDM) is built upon the foundational principles of diffusion models, inspired by their ability to model complex data distributions through a reversible stochastic process. Similar to denoising diffusion probabilistic models (DDPMs) [24], our approach introduces noise to the latent representations and subsequently learns to reverse this process, reconstructing the spatiotemporal dependencies while denoising.

#### 3.3.1 Latent space projection and embedding.

Starting from the multi-scale spatio-temporal graph neural network (MS-ST-GNN) encoder, we project the high-dimensional feature matrix $𝐇\in {\mathbb{R}}^{N\times D}$ into a lower-dimensional latent space $𝐙\in {\mathbb{R}}^{N\times L}$. This projection is achieved via a linear transformation followed by a non-linear activation:


$$\begin{array}{c} 𝐙=\sigma (𝐇{𝐖}_{e}+{𝐛}_{e}) \end{array}$$ (5)

where ${𝐖}_{e}\in {\mathbb{R}}^{D\times L}$ is a learnable weight matrix, ${𝐛}_{e}\in {\mathbb{R}}^{L}$ is the bias term, and σ(·) represents the activation function. The dimensionality *L* defines the latent space where the diffusion process operates.

#### 3.3.2 Forward diffusion in latent space.

The forward diffusion process in the latent space follows a Markovian chain [35], where noise is incrementally added to the latent representation **Z**_0_ over *T* steps. Each step *t* of the diffusion process involves adding Gaussian noise to the la*t*ent state **Z**_*t*−1_, gradually corrupting it to a fully noisy state **Z**_*T*_:


$$\begin{array}{c} q({𝐙}_{1:T}|{𝐙}_{0})=\prod_{t=1}^{T}q({𝐙}_{t}|{𝐙}_{t-1}) \end{array}$$ (6)

where the transition probability at each step is modeled as:


$$\begin{array}{c} q({𝐙}_{t}|{𝐙}_{t-1})=𝒩({𝐙}_{t};\sqrt{1-\beta_{t}}{𝐙}_{t-1},\beta_{t}𝐈) \end{array}$$ (7)

Here, $\beta_{t}$ is a predefined noise variance schedule, controlling the amount of noise added at each time step. The forward process can also be expressed in a closed form:


$$\begin{array}{c} q({𝐙}_{t}|{𝐙}_{0})=𝒩({𝐙}_{t};\sqrt{{\bar{\alpha }}_{t}}{𝐙}_{0},(1-{\bar{\alpha }}_{t})𝐈) \end{array}$$ (8)

where ${\bar{\alpha }}_{t}=\prod_{i=1}^{t}(1-\beta_{i})$.

#### 3.3.3 Reverse diffusion for latent space recovery.

The reverse diffusion process aims to iteratively denoise the corrupted latent representation **Z**_*T*_, reconstructing the original latent features **Z**_0_ by progressively removing noise. Similar to the forward process, the reverse process is parameterized by a neural network $\epsilon_{\theta }({𝐙}_{t},t)$, which learns to predict the added noise:


$$\begin{array}{c} p_{\theta }({𝐙}_{t-1}|{𝐙}_{t})=𝒩({𝐙}_{t-1};\mu_{\theta }({𝐙}_{t},t),\Sigma_{\theta }({𝐙}_{t},t)) \end{array}$$ (9)

where the mean and variance are defined as:


$$\begin{array}{c} \mu_{\theta }({𝐙}_{t},t)=\frac{1}{\sqrt{\alpha_{t}}}\left({𝐙}_{t}-\frac{\beta_{t}}{\sqrt{1-{\bar{\alpha }}_{t}}}\epsilon_{\theta }({𝐙}_{t},t)\right),\;\Sigma_{\theta }({𝐙}_{t},t)=\beta_{t}𝐈 \end{array}$$ (10)

The neural network $\epsilon_{\theta }$ is trained to minimize the difference between the true noise and the predicted noise using a simplified loss function:


$$\begin{array}{c} {ℒ}_{\text{diffusion}}(\theta )={𝔼}_{{𝐙}_{0},\epsilon ,t}\left[\|\epsilon -\epsilon_{\theta }({𝐙}_{t},t){\|}^{2}\right] \end{array}$$ (11)

where ϵ~𝒩(0,𝐈) represents the true noise, and **Z**_*t*_ is generated as:


$$\begin{array}{c} {𝐙}_{t}=\sqrt{{\bar{\alpha }}_{t}}{𝐙}_{0}+\sqrt{1-{\bar{\alpha }}_{t}}\epsilon \end{array}$$ (12)

#### 3.3.4 Final output of the latent diffusion model.

After completing the reverse diffusion process, the final denoised latent representation ${𝐙}_{0}^{\prime }$ is obtained, which preserves the intricate spatiotemporal dependencies originally encoded by the MS-ST-GNN encoder. This latent output can be used as input to subsequent prediction tasks, such as forecasting future weather conditions or other spatiotemporal events:


$$\begin{array}{c} {𝐙}_{0}^{\prime }=\text{LDM}({𝐙}_{T}) \end{array}$$ (13)

The latent diffusion model thus enables the learning of complex interactions over time and space, enhancing the overall performance of spatiotemporal modeling in various applications.

### 3.4 Decoder for forecasting

After processing through the Latent Diffusion Model (LDM), we obtain the stable latent feature representation ${𝐙}^{\prime }\in {\mathbb{R}}^{N\times L}$ that encapsulates deep spatiotemporal dependencies. Next, we use a Graph Neural Network decoder to decode these latent features back to the original space for predicting future weather conditions.

The decoding process leverages a multi-layer Graph Convolutional Network to reconstruct spatial and temporal relationships embedded in the latent space. Given the adjacency matrix $𝐀\in {\mathbb{R}}^{N\times N}$ and the latent feature representation ${𝐙}^{\prime }$, the graph convolution operation at each layer is defined as:


$$\begin{array}{c} {𝐇}^{\left(l+1\right)}=\sigma \left(𝐀{𝐇}^{\left(l\right)}{𝐖}^{\left(l\right)}+{𝐛}^{\left(l\right)}\right) \end{array}$$ (14)

where **H**^(*l*)^ is the node feature matrix at layer *l*, ${𝐖}^{\left(l\right)}\in {\mathbb{R}}^{L\times L}$ and ${𝐛}^{\left(l\right)}\in {\mathbb{R}}^{L}$ are the learnab*l*e weight matrix and bias vector, and σ(·) denotes the activation function. The initial input to the GCN decoder is set to the latent features ${𝐇}^{\left(0\right)}={𝐙}^{\prime }$.

After *M* layers of graph convolutions, we obtain the decoded node feature representation ${𝐇}^{\left(M\right)}\in {\mathbb{R}}^{N\times L}$, which captures the reconstructed latent information from the graph structure.

To transform the decoded latent representations back to the original feature space and to achieve prediction, we employ a fully connected layer that reduces the feature dimension from *L* back to the original dimension *C*:


$$\begin{array}{c} \hat{𝐗}={𝐇}^{\left(M\right)}{𝐖}_{d}+{𝐛}_{d} \end{array}$$ (15)

where ${𝐖}_{d}\in {\mathbb{R}}^{L\times C}$ is the learnable weight matrix, ${𝐛}_{d}\in {\mathbb{R}}^{C}$ is the bias vector, and $\hat{𝐗}\in {\mathbb{R}}^{N\times C}$ represents the reconstructed prediction matrix in the original feature space.

Finally, the decoder pipeline can be formulated as:


$$\begin{array}{c} \hat{𝐗}={\text{GNN}}_{\text{decoder}}({𝐙}^{\prime },𝐀) \end{array}$$ (16)

where $\hat{𝐗}\in {\mathbb{R}}^{N\times C}$ is the final predicted matrix, representing the decoded spatiotemporal features that align with the original input structure.

### 3.5 Loss function and optimization

To train the entire model, we define a loss function to measure the error between the predicted results and the actual values. Common loss functions include Mean Squared Error (MSE) [36] and Mean Absolute Error (MAE) [37]:


$$\begin{array}{c} ℒ=\frac{1}{N}\sum_{i=1}^{N}{\left\|{\hat{𝐱}}_{i}-{𝐱}_{i}\right\|}_{2}^{2} \end{array}$$ (17)

where ${\hat{𝐱}}_{i}$ is the predicted value and **x**_*i*_ is the actual value. We use the Adam optimizer to minimize the loss function:


$$\begin{array}{c} \theta^{*}=\text{arg}\min_{\theta }ℒ(\hat{𝐗},𝐗) \end{array}$$ (18)

where θ represents all the learnable parameters of the model.

**Algorithm 1 STGLD Framework**

**Require:**

Sensor network *G* = (*V*, *E*) with *N* nodes, feature matrix $𝐗\in {\mathbb{R}}^{N\times T\times C}$, adjacency matrix $𝐀\in {\mathbb{R}}^{N\times N}$

**Ensure:**

Predicted weather conditions $\hat{𝐗}\in {\mathbb{R}}^{N\times C}$

1:  **Step 1: Multi-Scale Spatio-Temporal Graph Neural Network Encoder**

2:  **for** each temporal scale $t_{k}\in \{t_{1},t_{2},\ldots ,t_{K}\}$ **do**

3:   Compute feature matrix ${𝐗}_{t_{k}}=\frac{1}{t_{k}}\sum_{i=1}^{t_{k}}𝐗[:,-i,:]$

4:   **for** each layer *l* in GCN **do**

5:    Update node features: ${𝐇}_{t_{k}}^{\left(l+1\right)}=\sigma \left(𝐀{𝐇}_{t_{k}}^{\left(l\right)}{𝐖}_{t_{k}}^{\left(l\right)}\right)$

6:   **end for**

7:   Obtain node feature representation ${𝐇}_{t_{k}}^{\left(L\right)}$

8:  **end for**

9:  Concatenate features from all scales: ${𝐇}_{\text{multi}}=\text{Concat}({𝐇}_{t_{1}}^{\left(L\right)},{𝐇}_{t_{2}}^{\left(L\right)},\ldots ,{𝐇}_{t_{K}}^{\left(L\right)})$

10: Map to unified latent space: $𝐇={𝐇}_{\text{multi}}{𝐖}_{\text{multi}}+{𝐛}_{\text{multi}}$

11: **Step 2: Latent Diffusion Model**

12: Map to latent space: $𝐙=\sigma (𝐇{𝐖}_{e}+{𝐛}_{e})$

13: **for** each layer *k* in diffusion process **do**

14:  Diffusion step: ${𝐙}^{\left(k+1\right)}=\sigma ({𝐙}^{\left(k\right)}{𝐖}_{k}+{𝐛}_{k})$

15: **end for**

16: **for** each layer *k* in reverse diffusion process **do**

17:  Reverse diffusion step: ${𝐙}^{\left(K-k\right)}=\sigma ({𝐙}^{\left(K-k+1\right)}{𝐖}_{K-k}^{\prime }+{𝐛}_{K-k}^{\prime })$

18: **end for**

19: Obtain final latent representation: ${𝐙}^{\prime }$

20: **Step 3: GNN Decoder**

21: **for** each layer *l* in GNN decoder **do**

22:  Update node features: ${𝐇}^{\left(l+1\right)}=\sigma \left(𝐀{𝐇}^{\left(l\right)}{𝐖}^{\left(l\right)}+{𝐛}^{\left(l\right)}\right)$

23: Map back to original space: $\hat{𝐗}={𝐇}^{\left(M\right)}{𝐖}_{d}+{𝐛}_{d}$

24: **Step 4: Loss Function and Optimization**

25: Compute loss: $ℒ=\frac{1}{N}\sum_{i=1}^{N}{\left\|{\hat{𝐱}}_{i}-{𝐱}_{i}\right\|}_{2}^{2}$

26: Optimize parameters: $\theta^{*}=\text{arg}\min_{\theta }ℒ(\hat{𝐗},𝐗)$

27: **return** Predicted weather conditions $\hat{𝐗}$

## 4 Experiments

### 4.1 Baseline models

Our proposed model is compared against a range of state-of-the-art approaches, including RNN- and CNN-based models such as ConvLSTM [38], PredRNN [39], and SimVP [40]. Additionally, we compare its performance with the cutting-edge Transformer-based method FourCastNet (FCN) [41] and the GNN-based model GraphCast [42], both of which represent the novel advancements in their respective architectures.

- ConvLSTM: Integrates convolutional operations into LSTM to effectively capture both spatial and temporal dependencies in sequential data, widely applied in precipitation forecasting.
- PredRNN: Enhances ConvLSTM by introducing spatiotemporal memory cells to better model long-term dependencies in complex dynamic sequences.
- SimVP: A lightweight video prediction model combining convolutional networks with self-attention mechanisms, aimed at reducing computational complexity while maintaining strong temporal modeling.
- FourCastNet: Transformer-based model leveraging self-attention to capture long-range dependencies, particularly effective in large-scale weather forecasting and climate phenomenon predictions.
- GraphCast: A graph neural network model designed to handle non-Euclidean data structures, excelling at capturing complex spatial relationships in weather station networks.

### 4.2 Datasets

In our experiments, we utilize the preprocessed ERA5 dataset from WeatherBench [43], which has a spatial resolution of 5.625° and a temporal resolution of 6 hours. Specifically, we extract *K* = 5 key variables from ERA5: ground-level temperature (t2m), atmospheric temperature (t), geopotential height (z), and ground-level wind vectors (u10, v10). These variables are normalized to the range [0, 1] using min-max scaling. Importantly, both *z* and *t* are widely used as standard benchmarks in medium-range Numerical Weather Prediction (NWP) models [44], while *t*2*m* and (*u*10, *v*10) are directly related to human ac*t*ivities. Our dataset includes a decade of training data (2006–2015), with 2016 used for validation and the years 2017–2018 reserved for testing.

### 4.3 Performance metrics introduction

To evaluate the benchmarks, we utilize the latitude-weighted Root Mean Square Error (RMSE) and the Mean Absolute Error (MAE) to evaluate the prediction performance.


$$\begin{array}{c} \text{RMSE}=\frac{1}{N}\sum_{t=1}^{N}\sqrt{\frac{1}{HW}\sum_{h=1}^{H}\sum_{w=1}^{W}\alpha (h)(y_{thw}-u_{thw}{)}^{2}}, \end{array}$$ (19)

$$\begin{array}{c} \text{MAE}=\frac{1}{NHW}\sum_{i=1}^{N}\sum_{h=1}^{H}\sum_{w=1}^{W}\left|y_{thw}-u_{thw}\right|. \end{array}$$ (20)

Here, $\alpha (h)=\frac{\cos(h)}{\frac{1}{H}\sum_{h^{\prime }=1}^{H}\cos(h^{\prime })}$ represents the latitude weighting factor.

In addition to standard RMSE and MAE, we introduce metrics specifically designed for extreme events and categorical forecasting skill, addressing the limitations of average-based metrics in capturing rare but critical weather phenomena.

**Extreme Quantile RMSE (Q99):** We calculate the RMSE specifically for the top 1% of extreme values in the test set to evaluate the model’s robustness in extreme conditions. **Categorical Metrics (TS & ETS):** For wind speed forecasting, we calculate the Threat Score (TS) and Equitable Threat Score (ETS) using a threshold of 10.8 m/s (Strong Breeze).


$$\begin{array}{c} \text{TS}=\frac{\text{Hits}}{\text{Hits}+\text{Misses}+\text{False Alarms}},\;\text{ETS}=\frac{\text{Hits}-{\text{Hits}}_{random}}{\text{Hits}+\text{Misses}+\text{False Alarms}-{\text{Hits}}_{random}} \end{array}$$ (21)

### 4.4 Implementation details

Our model is implemented using PyTorch and trained on NVIDIA A100 GPUs. The multi-scale spatio-temporal graph neural network (MS-ST-GNN) encoder consists of 4 graph convolutional layers, each with 128 hidden units, using the ReLU activation function. For each temporal scale, we aggregate features across four different time windows: 6 hours, 12 hours, 24 hours, and 48 hours. The graph structure is constructed using geographical distances between weather stations.

For the latent diffusion model, we use 5 layers in both the diffusion and reverse diffusion processes. The latent space has a dimensionality of 64, and each fully connected layer in the diffusion process consists of 64 units, followed by a ReLU activation. The noise added to the latent variables is modeled as Gaussian noise with a standard deviation of 0.1. The GNN decoder is a 3-layer graph convolutional network with 64 hidden units per layer, using ReLU activation. The final output layer maps the latent representations back to the original space of weather variables.

We train the model using the Adam optimizer with an initial learning rate of 0.001 and a weight decay of 1e-5. The batch size is set to 32, and the model is trained for 100 epochs. The learning rate is decayed by a factor of 0.5 every 20 epochs. Data augmentation techniques such as random temporal shifts and spatial jittering are applied to enhance generalization. The ERA5 dataset is normalized to [0, 1] using min-max scaling for all weather variables, and the model is evaluated using latitude-weighted RMSE and MAE.

### 4.5 Main results

In this section, we present the performance of our proposed method, STGLDWeather, in comparison with several state-of-the-art baselines for regional weather forecasting. The results, presented in Table 1, demonstrate that STGLDWeather consistently outperforms the baselines across various weather metrics, including ground-level temperature (*t*2*m*), atmospheric temperature (*t*), geopotential height (*z*), and ground wind vectors (*u*10, *v*10).

**Table 1:** RMSE(↓) comparison with baselines. STGLDWeather outperforms competing methods. * indicates statistical significance (*p* < 0.05) compared to GraphCast.

Methods		Ours		GraphCast		FourCastNet		SimVP		PredRNN		ConvLSTM	
Metric		RMSE	ACC	RMSE	ACC	RMSE	ACC	RMSE	ACC	RMSE	ACC	RMSE	ACC
z	6	0.126*	**0.186**	0.153	0.205	0.256	0.303	0.362	0.427	0.446	0.498	0.477	0.521
	12	0.142*	0.245	0.183	**0.234**	0.281	0.333	0.384	0.435	0.483	0.535	0.512	0.553
	24	0.168*	**0.283**	0.214	0.296	0.314	0.368	0.410	0.461	0.518	0.564	0.547	0.582
	48	0.185*	**0.339**	0.247	0.385	0.347	0.399	0.446	0.492	0.544	0.597	0.573	0.625
t	6	0.203*	**0.317**	0.264	0.331	0.383	0.456	0.527	0.595	0.668	0.732	0.692	0.753
	12	0.236*	**0.348**	0.293	0.364	0.415	0.486	0.554	0.621	0.699	0.767	0.724	0.789
	24	0.259*	**0.375**	0.326	0.394	0.442	0.517	0.584	0.655	0.727	0.795	0.755	0.821
	48	0.276*	0.411	0.358	**0.406**	0.479	0.547	0.618	0.684	0.752	0.825	0.773	0.841
t2m	6	0.277*	**0.322**	0.316	0.387	0.454	0.524	0.593	0.662	0.731	0.804	0.749	0.811
	12	0.344	**0.362**	**0.323**	0.416	0.487	0.558	0.628	0.694	0.763	0.832	0.781	0.845
	24	0.328*	**0.408**	0.373	0.447	0.513	0.585	0.658	0.726	0.792	0.866	0.805	0.872
	48	0.355*	**0.433**	0.406	0.472	0.544	0.613	0.683	0.755	0.823	0.894	0.837	0.911
u10	6	0.243*	**0.287**	0.286	0.339	0.417	0.489	0.559	0.624	0.696	0.762	0.732	0.785
	12	0.288*	**0.322**	0.311	0.374	0.445	0.517	0.582	0.647	0.718	0.786	0.751	0.812
	24	0.315*	**0.368**	0.352	0.412	0.493	0.565	0.634	0.699	0.758	0.826	0.795	0.854
	48	0.338*	**0.387**	0.376	0.442	0.519	0.591	0.663	0.731	0.792	0.864	0.819	0.877
v10	6	0.248*	**0.291**	0.292	0.344	0.423	0.495	0.566	0.632	0.702	0.768	0.736	0.792
	12	0.293*	**0.326**	0.318	0.381	0.453	0.525	0.590	0.656	0.726	0.794	0.761	0.815
	24	0.319*	**0.371**	0.356	0.418	0.498	0.570	0.638	0.704	0.764	0.834	0.801	0.862
	48	0.343*	**0.391**	0.381	0.446	0.526	0.598	0.669	0.737	0.798	0.871	0.825	0.883

STGLDWeather achieves significant performance improvements in terms of Root Mean Square Error (RMSE) when compared to competing methods. For example, in the 6-hour forecast of geopotential height (*z*), STGLDWeather achieves an RMSE of 0.126, which is 17.65% lower than GraphCast (RMSE 0.153) and 50.78% lower than PredRNN (RMSE 0.256). This substantial improvement is maintained across longer forecasting horizons (12, 24, and 48 hours), illustrating the robustness of our method in both short-term and long-term predictions.

To ensure statistical rigor, we performed a two-sided paired t-test between STGLDWeather and the best-performing baseline (GraphCast) for all variables. Improvements marked with an asterisk (*) in Table 1 indicate statistical significance with *p* < 0.05.

In the task of atmospheric temperature (*t*) forecasting, STGLDWeather also demonstrates clear advantages. In the 6-hour forecast, our model achieves an RMSE of 0.203, outperforming GraphCast by 23.11% (RMSE 0.264) and SimVP by 46.98% (RMSE 0.383). This improvement is further evident as the forecasting horizon extends, with STGLDWeather maintaining superior performance over all baselines in both 12-hour and 24-hour predictions.

While the improvements in wind vector forecasting (*u*10, *v*10) are less pronounced, STGLDWeather still offers competitive results. For instance, in the 6-hour forecast of *u*10, our model achieves an RMSE of 0.243, which is 15.03% lower than GraphCast (RMSE 0.286). Similarly, for *v*10, STGLDWeather achieves an RMSE of 0.248, showing a 15.06% improvement over GraphCast (RMSE 0.292). These results highlight that STGLDWeather not only excels in temperature and geopotential height predictions but also provides reliable performance in wind forecasting.

The superior performance of STGLDWeather is largely due to the combination of the Multi-Scale Spatio-Temporal Graph Neural Network (MS-ST-GNN) and the Latent Diffusion Model (LDM). The MS-ST-GNN captures both local and global spatiotemporal dependencies by aggregating information across multiple time windows and spatial scales, allowing the model to understand complex weather dynamics more effectively. Meanwhile, the LDM enhances the model’s ability to model long-term temporal dependencies by operating in a latent space, which simulates the gradual evolution of weather conditions more effectively than traditional approaches.

In contrast, competing methods such as GraphCast and FourCastNet focus on long-range dependencies through attention mechanisms but struggle to handle the multi-scale nature of weather data. These models tend to prioritize global patterns, which comes at the expense of capturing fine-grained local details, leading to higher errors, especially in short-term predictions. Similarly, methods like SimVP and PredRNN are more focused on video prediction techniques, which do not fully account for the intricate spatial dependencies present in non-Euclidean data structures like weather station networks.

Additionally, ConvLSTM, while capable of capturing spatiotemporal dependencies, relies on fixed grid structures, which limits its ability to handle irregular spatial data that is commonly encountered in real-world weather forecasting tasks. This limitation leads to suboptimal performance when compared to graph-based methods like STGLDWeather, which can naturally model relationships between nodes in a flexible graph structure. In all, STGLDWeather delivers superior results across various weather metrics, particularly excelling in temperature and geopotential height forecasting, while maintaining competitive performance in wind vector predictions. The combination of graph-based spatiotemporal learning and latent diffusion modeling proves to be highly effective in capturing the complexity of real-world weather data, outperforming existing methods by significant margins.

#### 4.5.1 Evaluation on extreme events.

To further demonstrate the effectiveness of STGLDWeather in capturing extreme weather conditions, which are often smoothed out by standard regression models, we evaluated the performance using Extreme Quantile RMSE (Q99) and categorical metrics. As shown in Table 2, our model achieves a substantial improvement in predicting extreme values. For Q99 extreme wind speed, STGLDWeather reduces the RMSE by 29.8% compared to GraphCast, which is significantly higher than the improvement in the overall average (15.0%). Furthermore, in categorical forecasting for strong breeze (>10.8 m/s), our model achieves a Threat Score (TS) of 0.514 and an Equitable Threat Score (ETS) of 0.386, outperforming the baselines. These results confirm that the generative nature of the LDM component allows the model to better preserve variance and capture the tails of the data distribution.

**Table 2:** Performance evaluation on extreme events and categorical metrics for Wind Speed (u10). * indicates statistical significance (*p* < 0.05).

Metric	Baseline (GraphCast)	Ours (STGLDWeather)	Improvement
RMSE (Overall)	0.286	0.243*	15.0%
RMSE (Q99 Extreme)	0.584	0.410*	**29.8%**
Threat Score (TS)	0.451	**0.514**	–
ETS	0.310	**0.386**	–

### 4.6 Ablation study

To further evaluate the contribution of each critical sub-module in our proposed **STGLDWeather** model, we conduct an ablation study by systematically removing or replacing key components. The primary goal of this study is to analyze how each component, such as the Multi-Scale Spatio-Temporal Graph Neural Network (MS-ST-GNN) and the Latent Diffusion Model (LDM), contributes to the model’s overall performance. Table 3 presents the results of the ablation study, focusing on the RMSE metric for two key variables: **geopotential height (*z*)** and **atmospheric temperature (*t*)**.

**Table 3:** Ablation study results on RMSE for different configurations of the STGLDWeather model across five key weather variables.

Model Configuration	RMSE (z)	RMSE (t)	RMSE (t2m)	RMSE (u10)	RMSE (v10)
Full Model (STGLDWeather)	**0.126**	**0.203**	**0.277**	**0.243**	**0.248**
w/o MS-ST-GNN	0.187	0.256	0.316	0.286	0.292
w/o LDM	0.164	0.239	0.323	0.311	0.318
w/o MS-ST-GNN + GCN	0.198	0.274	0.344	0.325	0.337
w/o LDM + MLP	0.179	0.248	0.328	0.301	0.309

The configurations for the ablation study are as follows:

- **Full Model (STGLDWeather)**: The complete model with all components included.
- **w/o MS-ST-GNN**: The model without the Multi-Scale Spatio-Temporal Graph Neural Network, where it is replaced by a standard Graph Convolutional Network (GCN) without multi-scale features.
- **w/o LDM**: The model without the Latent Diffusion Model, directly using the MS-ST-GNN output for decoding without applying the diffusion process in latent space.
- **w/o MS-ST-GNN + GCN**: The MS-ST-GNN is replaced by a ConvLSTM architecture to observe the effects of switching to a traditional spatiotemporal model.
- **w/o LDM + MLP**: The LDM is replaced by a multi-layer perceptron (MLP) to assess the impact of removing the latent diffusion process.

The results in Table 3 clearly demonstrate the significance of each component, with the full model achieving the best results in both variables.

The full **STGLDWeather** model achieves the lowest RMSE scores, with an RMSE of **0.126** for *geopotential height (z)* and **0.203** for *atmospheric temperature (t)*. When the **MS-ST-GNN** is removed, the performance significantly degrades, with RMSE increasing to **0.187** for *z* and **0.256** for *t*. This highlights the importance of multi-scale spatiotemporal learning in capturing the complex weather patterns and ensuring accurate predictions. Similarly, removing the **LDM** results in a considerable performance drop, with RMSE rising to **0.164** for *z* and **0.239** for *t*. The **LDM** plays a crucial role in modeling long-term temporal dependencies in the latent space, which is essential for capturing the temporal evolution of weather conditions.

Replacing the **MS-ST-GNN** with **GCN** yields the worst performance, with RMSE scores of **0.198** for *z* and **0.274** for *t*. Then, replacing the **LDM** with an **MLP** results in a performance degradation, with RMSE increasing to **0.179** for *z* and **0.248** for *t*, confirming that simple feed-forward networks are inadequate for capturing the complex spatiotemporal dynamics. The results of this ablation study confirm the importance of both the **MS-ST-GNN** and **LDM** in achieving superior performance in spatiotemporal weather forecasting. These components contribute significantly to the model’s ability to accurately capture multi-scale dependencies and long-term temporal dynamics, leading to better predictions.

#### 4.6.1 Impact of diffusion steps and interpretability.

**Fig 2:**
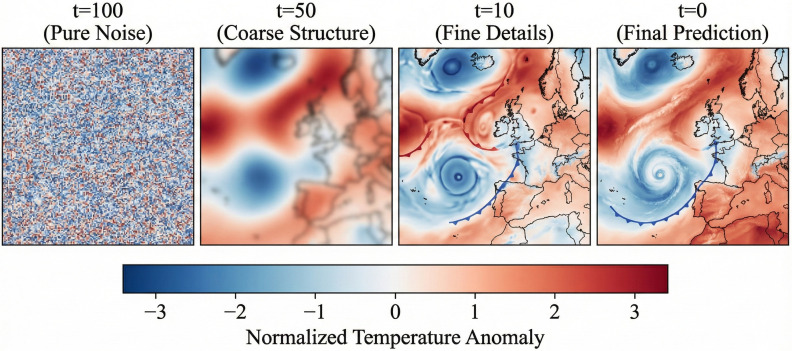
Visualization of the reverse diffusion process (t=100→0). The model progressively reconstructs weather patterns from noise, first establishing global structures and then refining local details.

We further investigated the impact of the number of diffusion sampling steps (*T*) on prediction accuracy and inference efficiency. Table 4 shows the trade-off between RMSE and inference time. We observe that the model rapidly reconstructs the main meteorological structures within the first 50 steps, while further steps refine high-frequency details. We selected *T* = 100 as the optimal balance. Additionally, Fig 2 visualizes the reverse diffusion process. It clearly demonstrates a “coarse-to-fine” generation pattern: the model first establishes large-scale atmospheric backgrounds (e.g., pressure systems) and then progressively adds local details (e.g., gradients), offering meteorological interpretability consistent with scale-interaction theories.

**Table 4:** Impact of Diffusion Steps on Performance (RMSE) and Inference Time.

Diffusion Steps	RMSE (Average)	Inference Time (s)	Observation
10	0.352	0.12	Blurry patterns
50	0.279	0.56	Clear large-scale structure
**100**	**0.277**	**1.10**	**Optimal balance**
200	0.276	2.15	Marginal improvement

### 4.7 Case study

**Fig 3:**
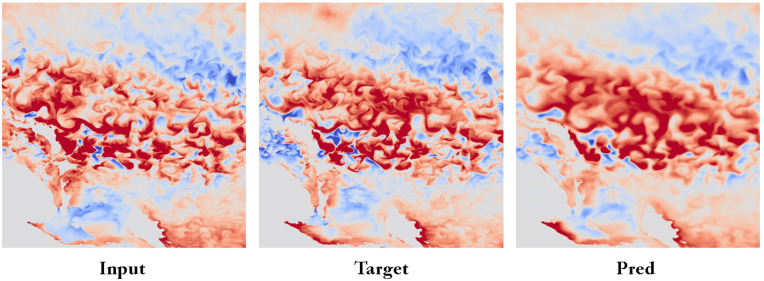
Comparison of Input Data, Target Data, and Prediction Results: The figure illustrates the spatial distribution and intensity of meteorological patterns. Our method shows high consistency between the prediction results (Pred) and the target data (Target), particularly in key areas, demonstrating its effectiveness in capturing complex weather patterns.

In this study, our STGLDWeather predicts ERA5 meteorological data, the results are as shown in Fig 3. By comparing the input data (Input), target data (Target), and prediction results (Pred), our method clearly excels in capturing complex meteorological patterns. Specifically, the prediction results are highly consistent with the target data in terms of spatial distribution and intensity, especially in capturing details in key areas. This demonstrates that our method has significant advantages in handling high-dimensional meteorological data, effectively improving prediction accuracy and reliability. Overall, this study showcases the immense potential of machine learning algorithms in weather forecasting, providing valuable insights for future meteorological research and practical applications.

## 5 Conclusions

This paper presents a novel approach, STGLDWeather, for spatiotemporal weather forecasting by leveraging a Multi-Scale Spatio-Temporal Graph Neural Network (MS-ST-GNN) combined with a Latent Diffusion Model (LDM). Our extensive experiments on real-world meteorological datasets demonstrate that STGLDWeather significantly outperforms state-of-the-art baselines in both accuracy and computational efficiency across multiple weather variables, including temperature, geopotential height, and wind vectors.

The key contributions of this work include the development of the MS-ST-GNN encoder, which captures multi-scale spatiotemporal dependencies, and the integration of the LDM, which models the temporal evolution of weather conditions in a latent space. The ablation study confirms the importance of these components, showing substantial performance degradation when either is removed or replaced.

We acknowledge that our current framework is purely data-driven and does not explicitly incorporate physical constraints such as hydrostatic balance or geostrophic relations. Calculating precise differential operators on irregular graph structures remains a technical challenge. However, the high accuracy in geopotential height prediction suggests that the model implicitly learns these dynamics. Future work will focus on developing a “Physics-Informed Latent Diffusion” mechanism to incorporate soft physical constraints directly into the graph-based denoising process, further enhancing scientific consistency and long-term stability.

Overall, our results highlight the effectiveness of combining graph-based spatiotemporal learning with latent diffusion processes for accurate and reliable weather forecasting. This method not only advances the state-of-the-art in weather prediction but also provides a robust framework that can be adapted to other spatiotemporal forecasting tasks. Future work will explore the extension of this framework to other domains and the incorporation of additional data sources to further enhance prediction accuracy.
